# Supplementary material for: Diagnostic Accuracy of Liquid Biopsy in Endometrial Cancer
Source: Cancers (Basel). 2021 Nov 16;13(22):5731. doi: 10.3390/cancers13225731 (PMC8616122; doi:10.3390/cancers13225731)
Supplement: Supplementary file 1 [file cancers-13-05731-s001.zip › cancers-1424460-supplementary.pdf]

cancers MDPI

Supplementary Materials: Diagnostic accuracy of liquid biopsy in endometrial cancer

Marta Łukasiewicz, Krzysztof Pastuszak, Sylwia Łapińska-Szumczyk, Robert Różański, Sjors G.J.G. In 't Veld, Michał Bieńkowski, Tomasz Stokowy, Magdalena Ratajska, Myron G. Best, Thomas Würdinger, Anna J. Żaczek, Anna Supernat, Jacek Jassem

Tables

Table S1. Gene panel summary. Genes covered in panel for targeted sequencing.

Link:
https://docs.google.com/spreadsheets/d/1qDc3VQg64OC_sPz6EN7Ibzc0xJ9fdeC_/edit?usp=sharing&ouid=103983408576558021742&rtpof=true&sd=true

Table S2. Description of the feature engineering used for Random Forest Classifier: We created and compared several feature sets that differed in the granularity of information about mutations as well as ways that this information was transformed. The basic sets contain information about the presence or absence of mutations in the relevant genes (set called "Gene") or are based on information about the severity of mutations, encoded in Variant Effect Predictors (sets called "VEP")[1]. The VEP scores have four possible values: LOW, MODERATE, HIGH and MODIFIER, the information provided in GDC. The first three values reflect the severity of mutation (e.g. a frameshift mutation will have a HIGH score, while missense mutations MODERATE). The last value is assigned to mutations of unknown significance. In total, five feature sets based on VEP scores were produced. To improve the classification performance, we also used two additional approaches to enrich the data with external information about the functional context in which the genes operate. In the first approach, we used Gene Ontology (GO, http://geneontology.org/) to map information about mutations to respective functional groups, at different levels of abstraction[2]. In the second approach, we used The SIGnaling Network Open Resource (SIGNOR, http://signor.uniroma2.it) – signaling models of oncogenesis to aggregate information about mutations along the relevant pathways[3.]

Link:
https://docs.google.com/spreadsheets/d/1vTkzAa-BIN-yVTG6C_xw6nd4JIjcbP1r/edit?usp=sharing&ouid=103983408576558021742&rtpof=true&sd=true

Table S3. Ranges of Extremaly Randomized Trees (ERT) hyperparameters values used in this study. The first 3 hyperparameters control the size of the individual trees. The max. features hyperparameter controls the number of features used in training of individual trees: if it is 1.0 then all features are used by every tree, otherwise a random sample is drawn for each tree independently, with the sample size equal to sqrt(nfeatures) or log2(nfeatures). The n_estimators hyperparameter which controls the forest size, was kept at 100 in initial finetuning and was only optimized in the subsequent stages to limit the computional cost. The values of two bottom hyperparameters had been assigned without finetuning. The rest of the forest hyperparameters' values were kept at default Scikit-Learn values.

Link:
https://docs.google.com/spreadsheets/d/1DWVTUHkqGb933lVL5cg_1tTD1sJAtkoZ/edit?usp=sharing&ouid=103983408576558021742&rtpof=true&sd=true

Table S4. TEPs sample list. Samples used for platelet classification, where the imPlatelet classifier was used [1].

Link:
https://docs.google.com/spreadsheets/d/1PnOXjGQna83bHSiNrfZiXykAzA7hanli/edit?usp=sharing&ouid=103983408576558021742&rtpof=true&sd=true

Table S5. ctDNA and primary EC sample list. Samples used for ctDNA and primary tumor classification where Random Forest Classifier was used. The model training was based on the data of 519 patients with EC, downloaded from the Genomic Data Commons (GDC) Data Portal. Patients' characteristics are available in Table 2. The data were randomly split (80:20) into the cross-validation set (415) and the test set (104), using stratification to ensure equal proportion of EC types in these sets. We focused on mutations found in the same 71 genes covered by a commercial cancer gene panel used in vitro part.

Link:
https://docs.google.com/spreadsheets/d/1Sfrj3EwM3iS0OFv0RwUetE8krSOMsdj0/edit?usp=sharing&ouid=103983408576558021742&rtpof=true&sd=true

Figures

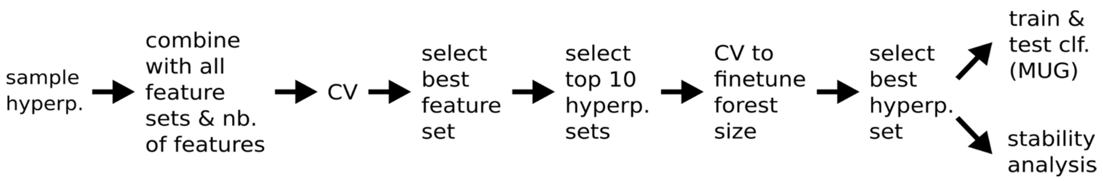

Figure S1. The stages of model development and testing. The top sequence shows the stages of model development, culminating in testing on MUG dataset. The bottom sequence shows what differences were made in testing on Bolivar et al. data. Bottom part shows which hyperparameters' values were selected at which stage.

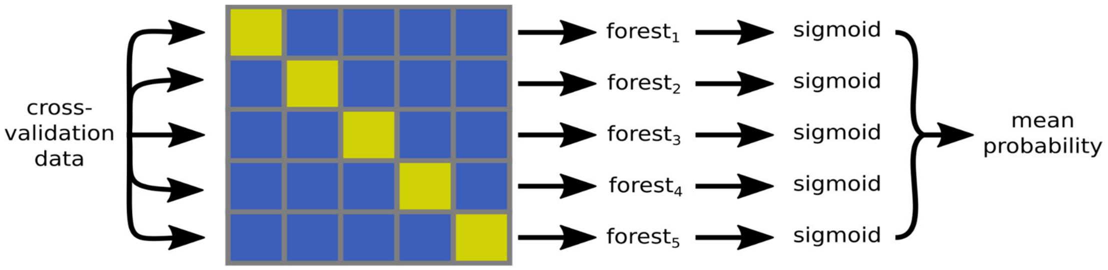

Figure S2. The structure of the final model: the final models were trained using a CalibratedClassifierCV meta-estimator from Scikit-Learn, with Extremely Randomized Trees (ERT) as base estimators. To train and calibrate the model, 5 ERT models were trained on different subsets of the crossvalidation data (blue boxes) and the remaining validation set (green boxes) was used for their calibration. The calibration was done using a sigmoid function. Predictions done by the model are average probabilities returned by the submodels.

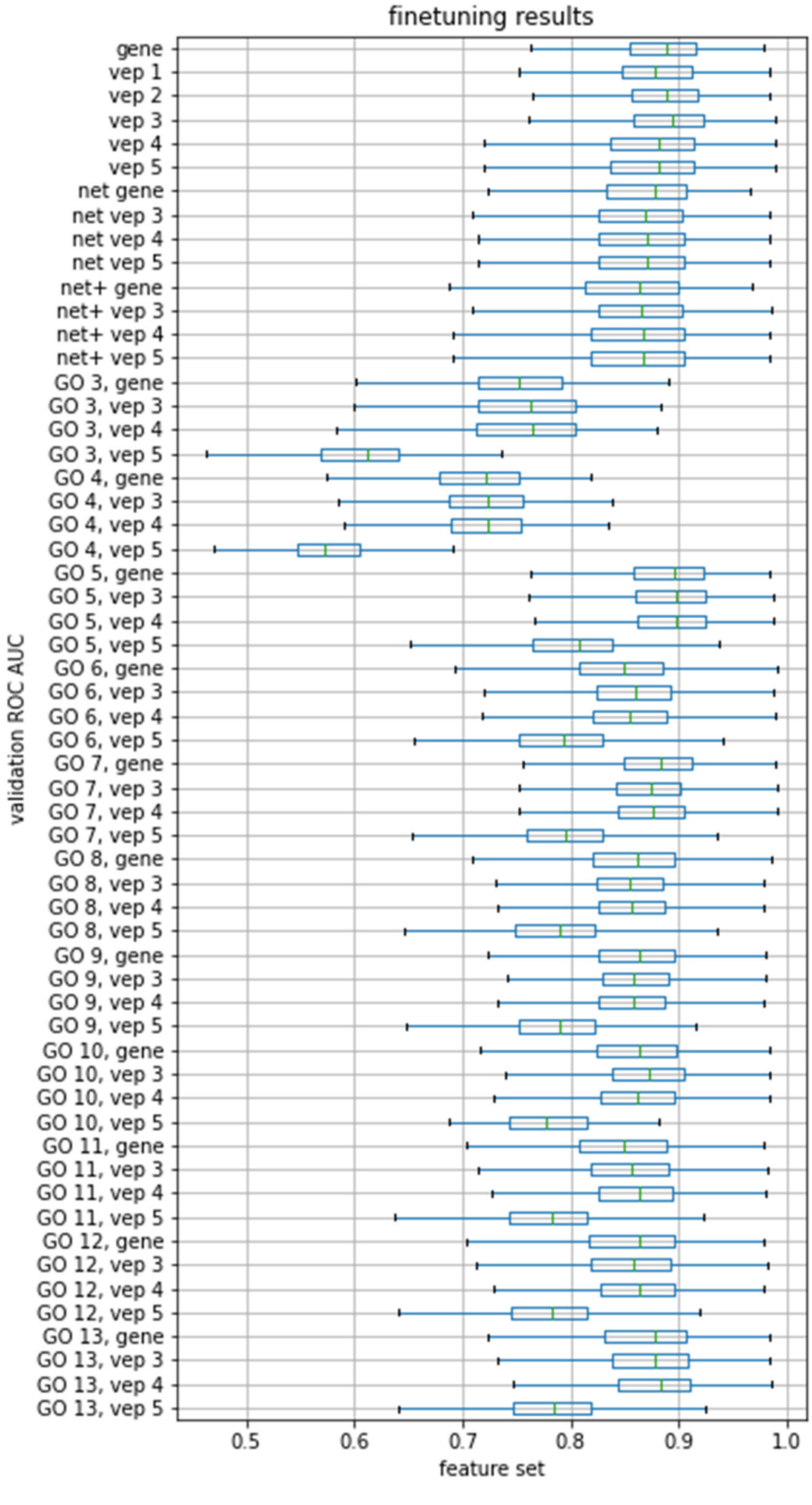

Figure S3. Performance of models on crossvalidation dataset, grouped by feature set. The results were obtained during the first finetuning stage (scheme presented above). The boundaries of the boxes show the first and third quartiles of the CV ROC AUC; the green line marks the median; the whiskers extend Table 1. 5*(Q3-Q1) or to the last datapoint if it is within that range. The best feature set (by first quartile, or equivalently 25th percentile) was GO 5, vep 4.

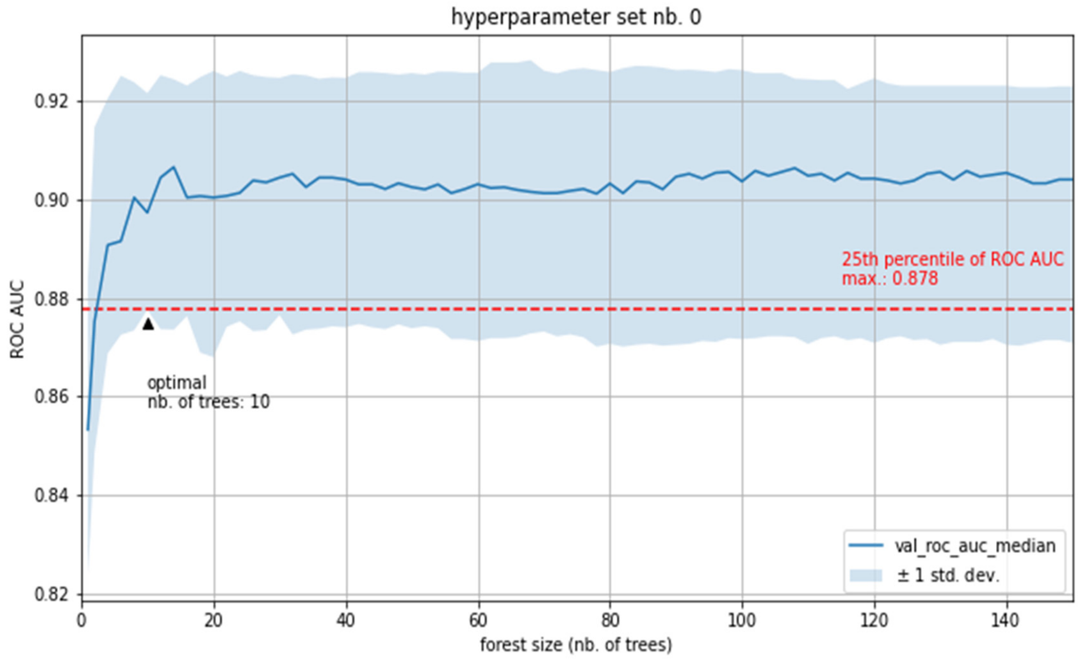

Figure S4. Results of forest size finetuning for the best hyperparameters set. The shaded region spans from the Figure S5. The quality of calibration of the final model on the test set (upper plots) and the crossvalidation set (bottom plots). Data were discretized into equally-sized buckets based on the predicted probability; for each bucket the fraction of positives (class 2) and mean probability was calculated and plotted.

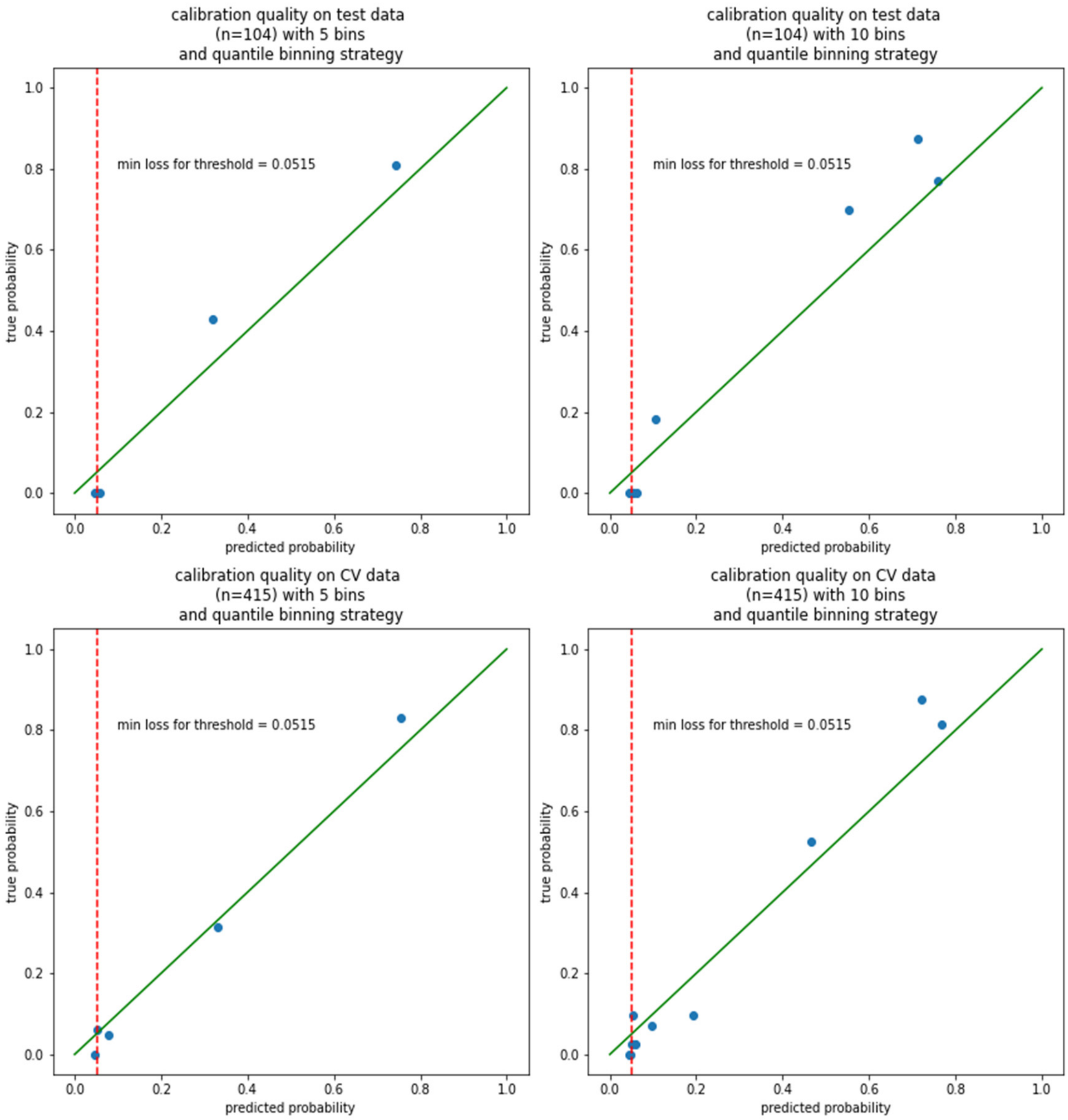

Figure S5. The quality of calibration of the final model on the test set (upper plots) and the crossvalidation set (bottom plots). Data were discretized into equally-sized buckets based on the predicted probability; for each bucket the fraction of positives (class 2) and mean probability was calculated and plotted.

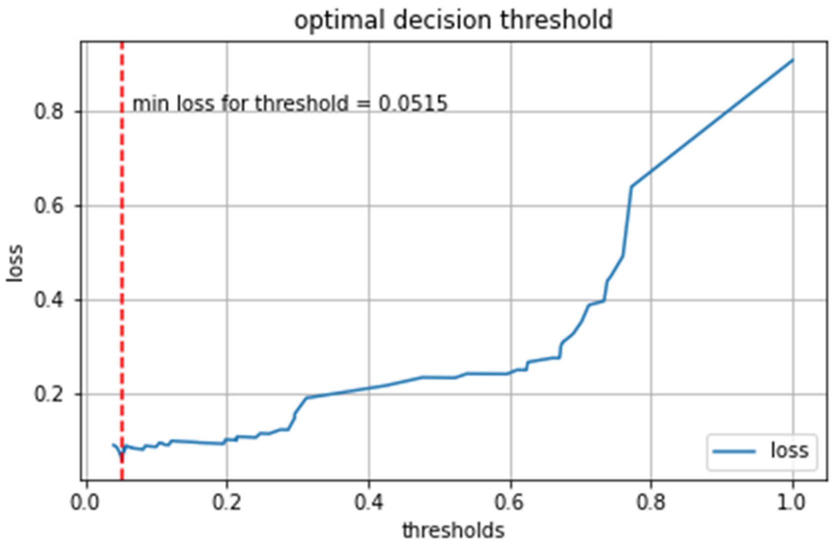

Figure S6. Results of decision threshold optimization. The threshold was optimised on the whole crossvalidation set.

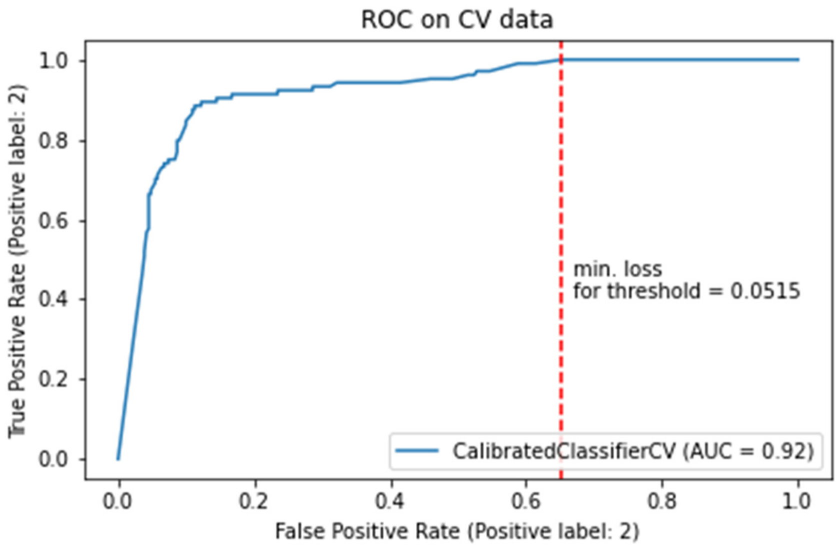

Figure S7. ROC curve for the crossvalidation data with the optimal decision threshold.

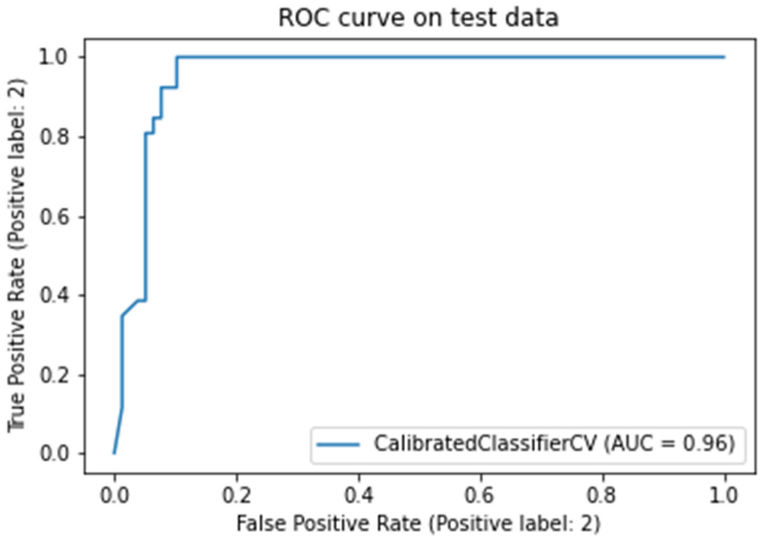

Figure S8. ROC curve for the test data.

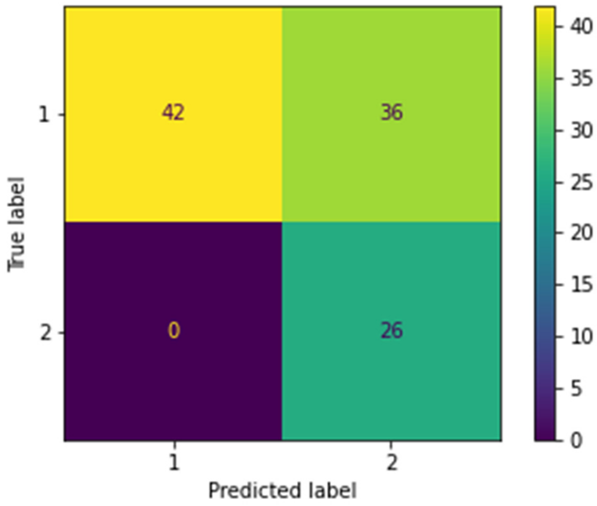

Figure S9. Confusion matrix for the test data and the optimal decision threshold.

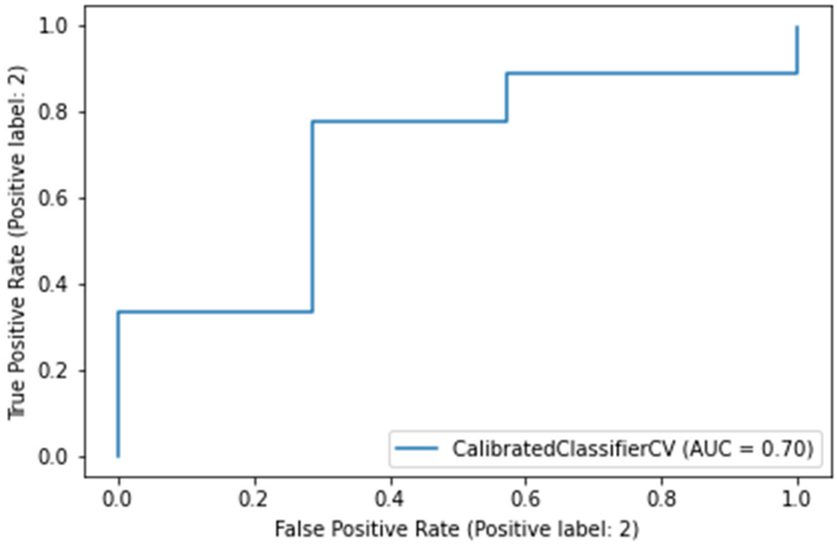

Figure S10. ROC curve for the MUG data.

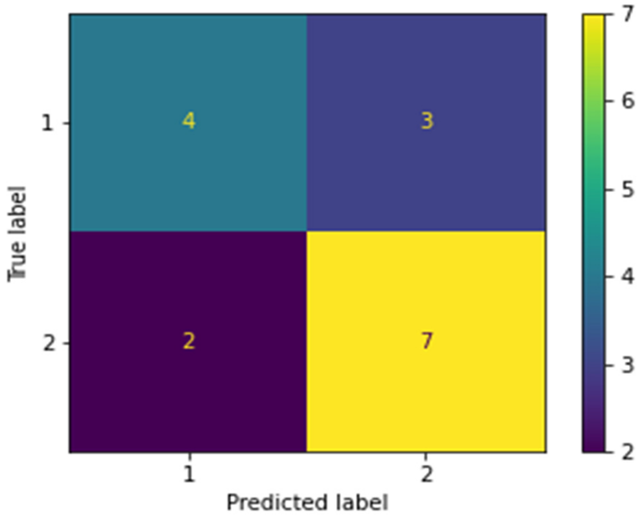

Figure S11. Confusion matrix for the MUG data and the optimal decision threshold.

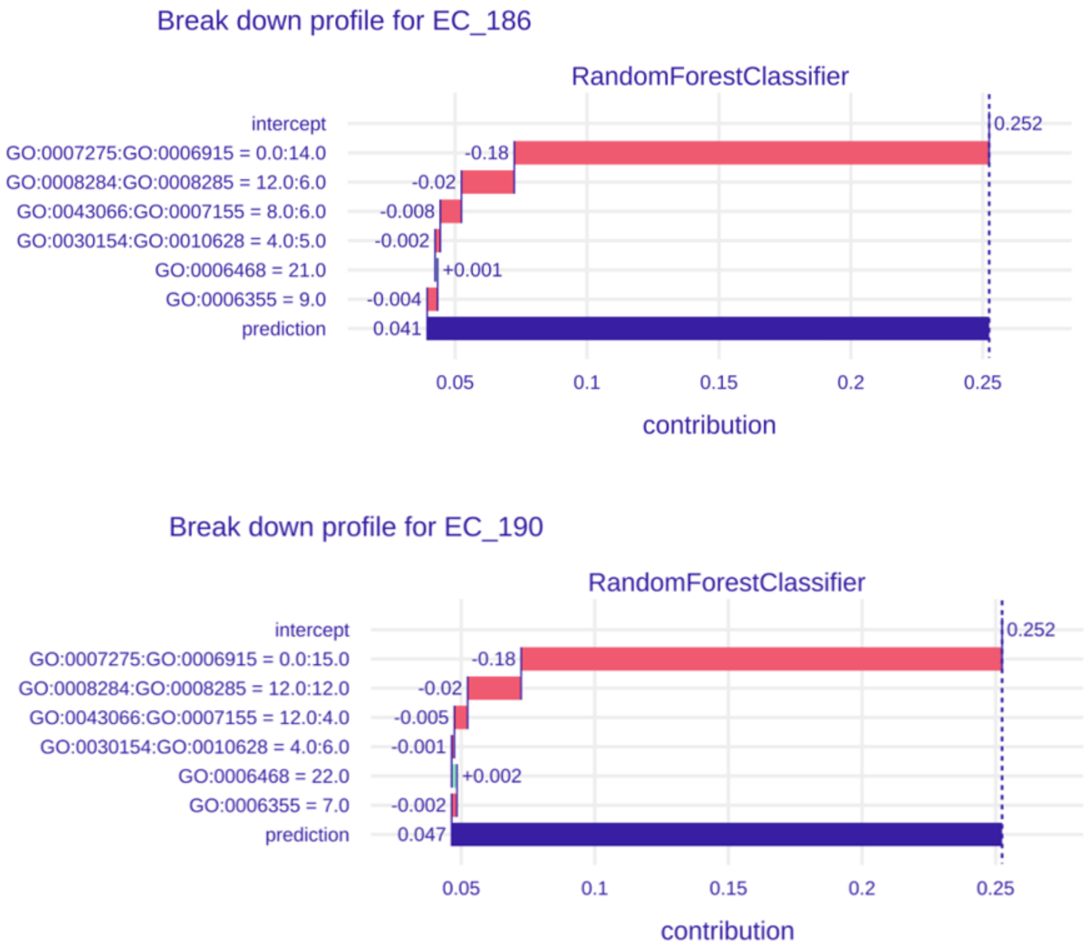

Figure S12. Breakdown plots for the MUG class 2 cases that were incorrectly classified by the final classifier.

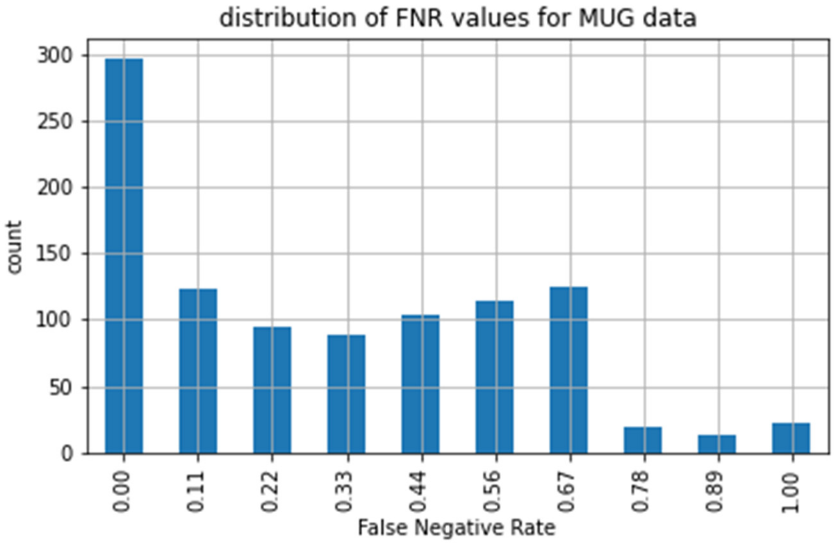

Figure S13. The results of the stability analysis: the distribution of False Negative Rate values on the MUG dataset.

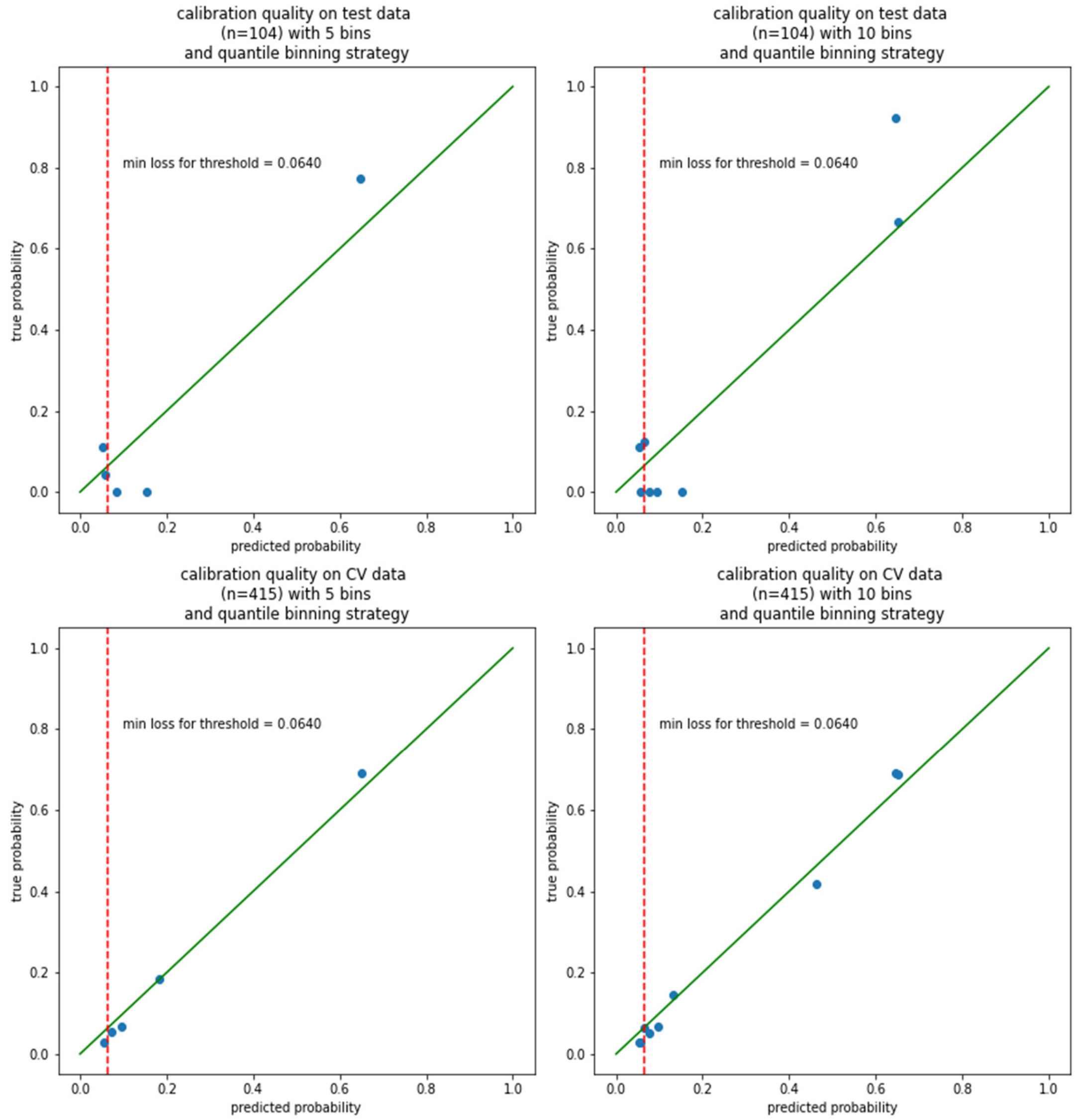

Figure S14. The quality of calibration of the final model on the test set (upper plots) and the crossvalidation set (bottom plots). Data were discretized into equally-sized buckets based on the predicted probability; for each bucket Table 2. and mean probability was calculated and plotted.

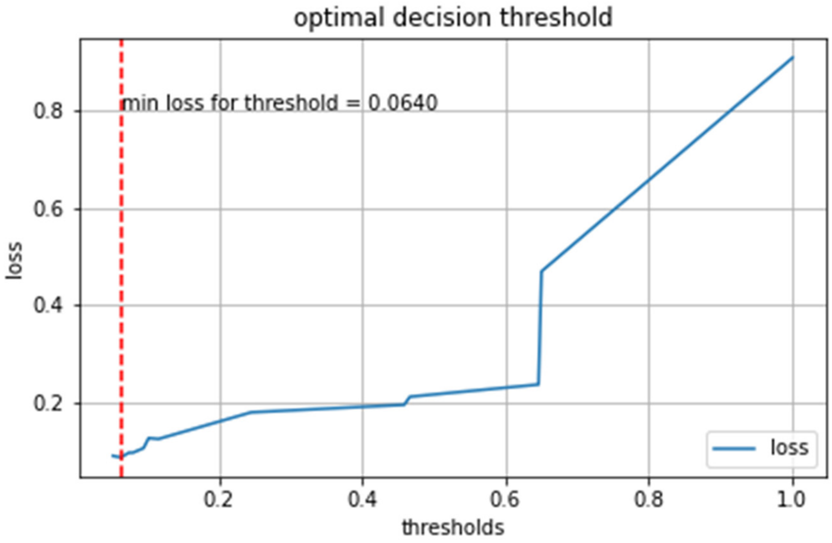

Figure S15. Results of decision threshold optimization. The threshold was optimized on the whole crossvalidation set.

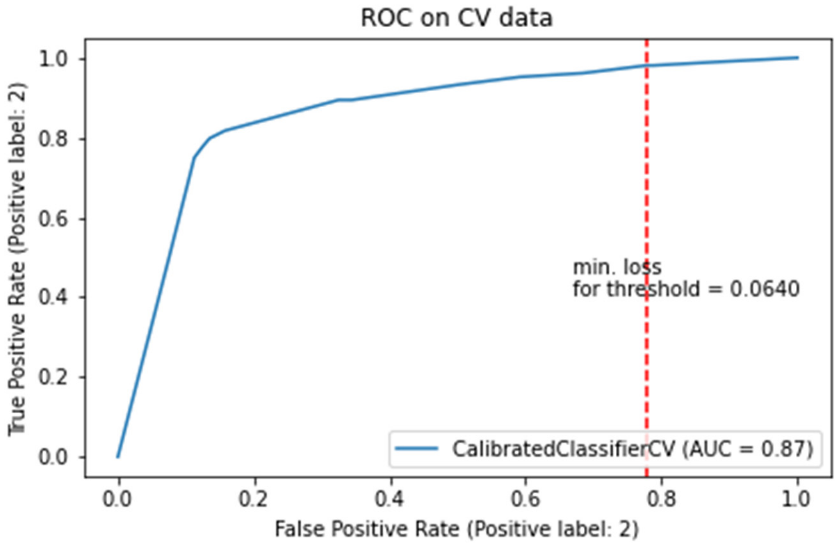

Figure S16. ROC curve for the crossvalidation data with the optimal decision threshold.

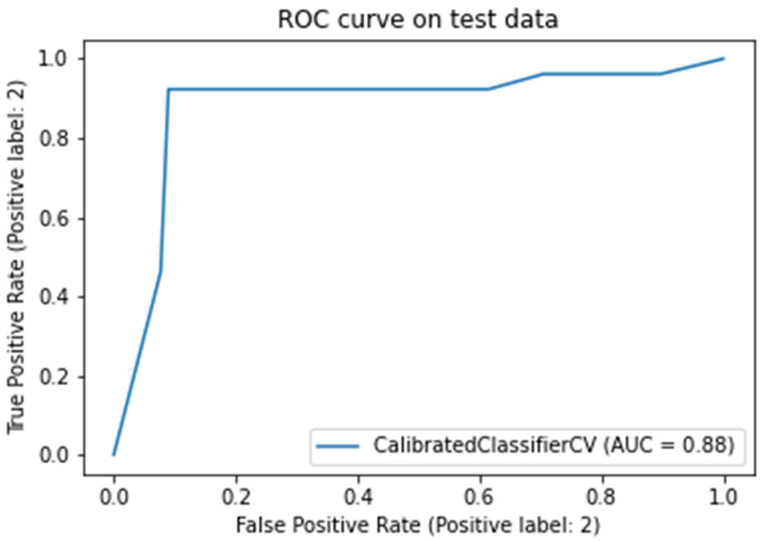

Figure S17. ROC curve for the test data.

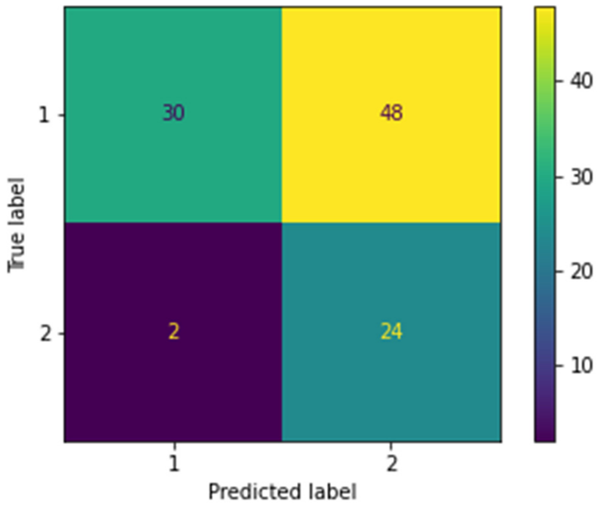

Figure S18. Confusion matrix for the test data and the optimal decision threshold.

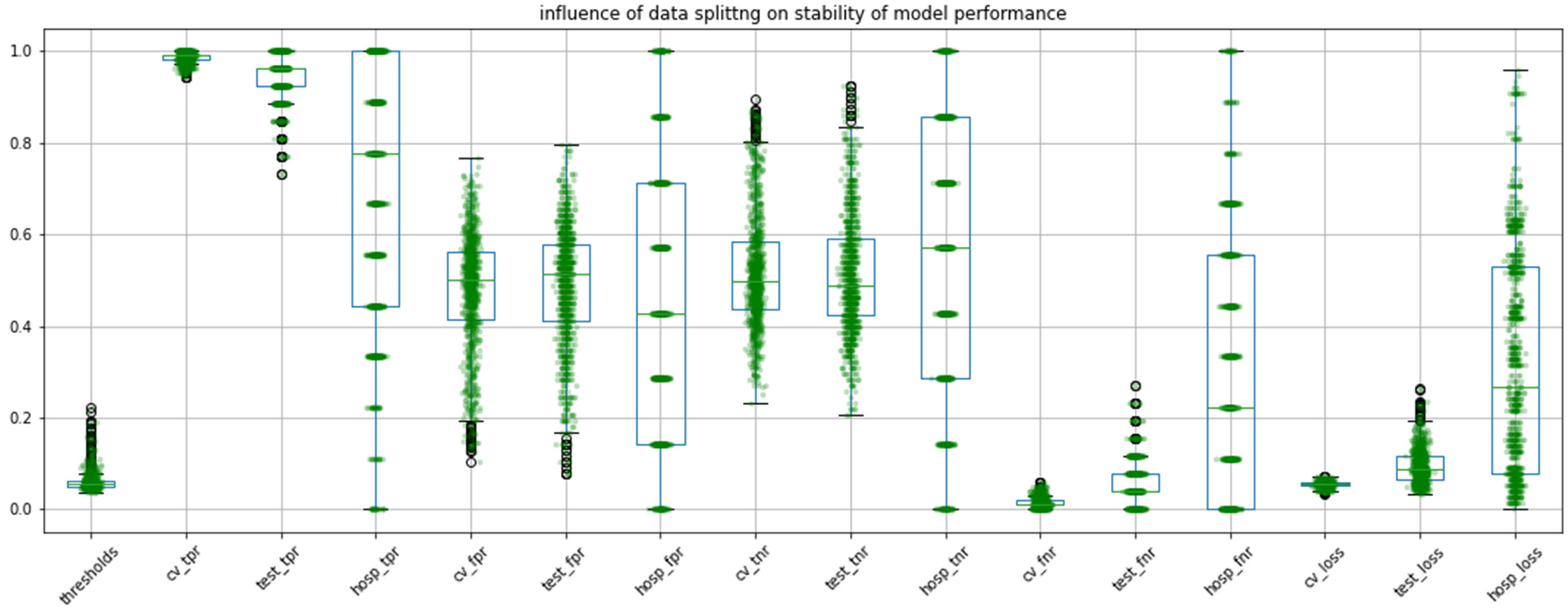

Figure S19. The results of the stability analysis: the results were obtained through repeated model training, calibration and testing, using different splits into test and crossvalidation data (1000 times in total); cv_ - scores on the full crossvalidation set; test_ - scores on the test set; hosp_ - scores on the MUG dataset; tpr - True Positive Rate; fpr - False Positive Rate; tnr - True Negative Rate; fnr - False Negative Rate.

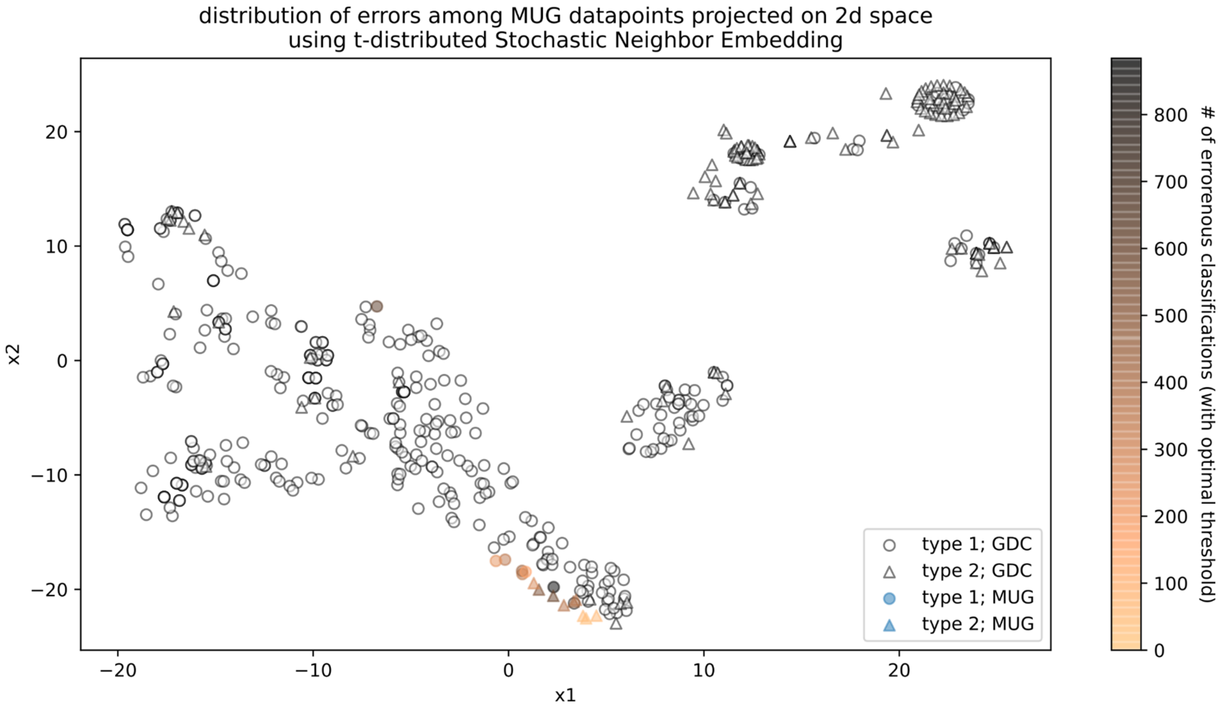

Figure S20. The results of the stability analysis: the distribution of errors among the MUG dataset; no errors are shown for the GDC dataset for the sake of clarity. The projection into 2D space was done using t-distributed Stochastic Neighbour Embedding.

References

1. McLaren, W.; Gil, L.; Hunt, S.E.; Riat, H.S.; Ritchie, G.R.S.; Thormann, A.; Flicek, P.; Cunningham, F. The Ensembl Variant Effect Predictor. *Genome Biol.* **2016** *17*, 122. https://doi.org/10.1186/s13059-016-0974-4.
2. Alterovitz, G.; Xiang, M.; Mohan, M.; Ramoni, M.F. GO PaD: the Gene Ontology Partition Database. *Nucleic Acids Res.* **2007**, *35*, D322–D327.
3. Perfetto, L.; Briganti, L.; Calderone, A.; Perpetuini, A.C.; Iannuccelli, M.; Langone, F.; Licata, L.; Marinkovic, M.; Mattioni, A.; Pavlidou, T.; *et al.* SIGNOR: a database of causal relationships between biological entities. *Nucleic Acids Res.* **2016**, *44*, D548–D554.
4. Bolivar, A. M. *et al.* Targeted next-generation sequencing of endometrial cancer and matched circulating tumor DNA: identification of plasma-based, tumor-associated mutations in early stage patients. *Mod. Pathol. Off. J. U. S. Can. Acad. Pathol. Inc* **2019**, *32*, 405–414.
